# Annotations

**Published:** 1895-02-09

**Authors:** 


### ANNOTATIONS.

### Cold: Healthy and Unhealthy.

IN England severe cold generally kills a good many people; in certain parts of Northern America cold still more severe puts new life into them. It requires no argumentation to show that there must be a definite reason for this. The chief reason is that English cold is mostly damp, whilst in North America it is mostly dry. There is the greatest difference in the world between dry air and damp air. The former is usually air pure and simple, possessing a full quantity of oxygen, and often charged highly with ozone. The latter consists of air mixed with the vapour of water. When the former air, fully oxygenated, is breathed, it stimulates more powerfully than champagne. The latter, less oxygenated, and charged with the vapour of water, not only does not stimulate, it depresses. The familiar fact of the winter fire burning brightly in frosty weather, and so dimly as to require constant stoking in dull and damp weather, is a homely argument, which the least scientific can understand. What is the practical application of these facts? It is, first, to make the air of living and sleeping apartments dry by means of fires, gas burners, oil stoves, and so on; and secondly, to protect the surface of the body against the effects of damp by means of abundance of dry, heat-retaining, and principally woollen clothing. Rapid movement of the body, as in quick walking or skating, is one of the most effectual preventives against chills, as also is the wearing of suitable caps or other head-clothing in apartments insufficiently warmed. The English climate, we have often said, is a good servant but a bad master. The first requisite towards making it a servant rather than a master is to thoroughly understand it, and to obey it in small points, in order that we may command it in great.

### Rural Sanitation and the Unemployed.

THE great economic problem of the day hinges on the growing mass of the unemployed. How the extension of the system of "water-carriage" of sewage touches this question in economics may not at first sight be apparent, but it becomes obvious when we consider the relation of the labourer to the land. To many the water-closet seems the acme of sanitary perfection and æsthetic nicety. So doubtless to the individual it is; to the community it is just the opposite, for the water-carriage of sewage on the one hand diminishes the openings for unskilled labour, on the other lessens the fertility of the land, lessens the food production of the country, introduces into every house appliances which if not kept in perfect order are the reverse of sanitary, and vastly increases the rates, and thus the rents, and thus the struggle for existence. This evil seems essential to large towns, but we cannot say that we regard with equanimity the growing encroachments of the water-closets in country districts where the land is starving for manure, and the labour which should be tilling it is starving too. It is obviously wrong that this evil should be allowed to go on increasing where land can still be had, where hamlets are developing into villages, villages into towns, and towns are stretching out their suburban tentacles far into the country. An ounce of practice is worth a pound of theory, and when a well-known sanitarian, Dr. Vivian Poore, in his "Rural Hygiene," tells us that for some years past he has been able to utilise the daily scavenging of twenty cottages, with an average population of at least one hundred persons, on a garden of one acre and a quarter in size, the whole secret of success being that the garden lies close to the cottages and that the refuse is removed every day, we cannot but see that the modern craze for drains and water-closets is far from being perfection, and where proper land can be had is absolutely wrong. Thus the town of the future should be widespread, and provided with sufficient garden space between the houses to utilise their refuse. Not only would the diseases due to overcrowding be thus diminished, but the town refuse, which at present is a cause of nuisance and expense, might perhaps by the constant employment of labour in its utilisation partly solve the problem of the unemployed.

### Methuselah: An Apologia.

MODERN medical science has something to say even in favour of the assertion that Methuselah lived to be 969 years old, and that something has been very well said by Dr. Samuel Wilks. The duration of life in warm-blooded animals for the most part bears a definite proportion to the duration of their immaturity. In other words, animals which rapidly become mature live short lives, whilst animals which mature more slowly attain to great ages. The dog, for example, is mature soon after completing his first year, say at the age of eighteen months. The human being does not reach the same stage of maturity in less than twenty-five years. Now the dog, if he is as well used as the human being, easily reaches the age of sixteen years. A rule of three sum will thus enable us to say with scientific reasonableness what the age of man ought to be, thus: $1\frac{1}{2} : 16 :: 25$ is to the age indicated by nature. That is to say, according to this rule, the full age of man ought to be not less than 266 years. "In reading of the great ages assigned to the early dwellers upon earth as given in the Bible," says Dr. S. Wilks, "it has never seemed to me an impossibility or an absurdity to suppose that human beings could reach a greater age than they do at the present day. Were it recorded that the early dwellers on the earth had reached their prime at the same time as we do at the present era, and then had lived a thousand years afterwards, I should have put the story aside as opposed to our knowledge of animal life. But it is very remarkable that the natural proportional stages of growth are preserved, the development being, of course, somewhat slower. The principal facts mentioned in the lives of the patriarchs, calculated to guide us, are the time of their marriage and the birth of their first child. Now if this be taken as the commencement of manhood, we see how this term is delayed, so that its proportion to the whole duration of life is preserved. Thus, Methuselah lived to be 969 years, and his first child Lamech was born when he was 187 years old." It is manifest that Dr. Wilks' remarks are very pertinent. Perhaps it will turn out some day that some other disputed facts of the Bible have as much to say for themselves as has been said by this scientific veteran on behalf of the claim made for Methuselah.

